# Detection and Characterization of Two Phenotypes of Candida parapsilosis in South Korea: Clinical Features and Microbiological Findings

**DOI:** 10.1128/spectrum.00066-23

**Published:** 2023-05-08

**Authors:** Jung-Hyun Byun, Eun Jeong Won, Hae Weon Cho, Daewon Kim, Hyukmin Lee, Soo Hyun Kim, Min Ji Choi, Seung A. Byun, Ga Yeong Lee, Seung-Jung Kee, Tae Yeul Kim, Mi-Na Kim, Jun Yong Choi, Dongeun Yong, Jong Hee Shin

**Affiliations:** a Department of Laboratory Medicine, Gyeongsang National University Hospital, Gyeongsang National University College of Medicine, Jinju, South Korea; b Department of Laboratory Medicine, Chonnam National University Medical School, Gwangju, South Korea; c Department of Laboratory Medicine, Asan Medical Center, University of Ulsan College of Medicine, Seoul, South Korea; d Department of Laboratory Medicine and Research Institute of Bacterial Resistance, Yonsei University College of Medicine, Seoul, South Korea; e Department of Laboratory Medicine, Myongji Hospital, Goyang, South Korea; f Department of Laboratory Medicine and Genetics, Samsung Medical Center, Sungkyunkwan University School of Medicine, Seoul, South Korea; g Department of Internal Medicine and AIDS Research Institute, Yonsei University College of Medicine, Seoul, South Korea; Agricultural Research Organization Volcani Center

**Keywords:** *Candida parapsilosis*, candidemia, fluconazole-nonsusceptible, sinking phenotype, Y132F *ERG11* mutation

## Abstract

We newly detected two (sinking and floating) phenotypes of Candida parapsilosis among bloodstream infection (BSI) isolates from Korean hospitals and assessed their microbiological and clinical characteristics. During the performance of a Clinical and Laboratory Standards Institute (CLSI) broth microdilution antifungal susceptibility testing, the sinking phenotype had a characteristic smaller button-like appearance because all yeast cells sank to the bottoms of the CLSI U-shaped round-bottom wells, whereas the floating phenotype comprised dispersed cells. Phenotypic analysis, antifungal susceptibility testing, *ERG11* sequencing, microsatellite genotyping, and clinical analysis were performed on C. parapsilosis isolates from 197 patients with BSI at a university hospital during 2006 to 2018. The sinking phenotype was detected in 86.7% (65/75) of the fluconazole-nonsusceptible (FNS) isolates, 92.9% (65/70) of the isolates harboring the Y132F *ERG11* gene substitution, and 49.7% (98/197) of all isolates. Clonality was more frequently observed for the Y132F-sinking isolates (84.6% [55/65]) than for all other isolates (26.5% [35/132]; *P < *0.0001). Annual incidence of Y132F-sinking isolates increased 4.5-fold after 2014, and two dominant genotypes, persistently recovered for 6 and 10 years, accounted for 69.2% of all Y132F-sinking isolates. Azole breakthrough fungemia (odds ratio [OR], 6.540), admission to the intensive care unit (OR, 5.044), and urinary catheter placement (OR, 6.918) were independent risk factors for BSIs with Y132F-sinking isolates. The Y132F-sinking isolates exhibited fewer pseudohyphae, a higher chitin content, and lower virulence in the Galleria mellonella model than the floating isolates. These long-term results illustrate the increasing BSIs caused by clonal transmission of the Y132F-sinking isolates of C. parapsilosis.

**IMPORTANCE** We believe that this is the first study describe the microbiological and molecular characteristics of bloodstream isolates of C. parapsilosis in Korea exhibiting two phenotypes (sinking and floating). An important aspect of our findings is that the sinking phenotype was observed predominantly in isolates harboring a Y132F substitution in the *ERG11* gene (92.9%), fluconazole-nonsusceptible (FNS) isolates (86.7%), and clonal BSI isolates (74.4%) of C. parapsilosis. Although the increase in the prevalence of FNS C. parapsilosis isolates has been a major threat in developing countries, in which the vast majority of candidemia cases are treated with fluconazole, our long-term results show increasing numbers of BSIs caused by clonal transmission of Y132F-sinking isolates of C. parapsilosis in the period with an increased echinocandin use for candidemia treatment in Korea, which suggests that C. parapsilosis isolates with the sinking phenotype continue to be a nosocomial threat in the era of echinocandin therapy.

## INTRODUCTION

Candida parapsilosis bloodstream infections (BSIs) caused by fluconazole-nonsusceptible (FNS) isolates have been increasingly reported worldwide (1, 2). Several recent studies indicate that the majority of FNS C. parapsilosis isolates harbor a Y132F substitution in the *ERG11* gene (referred to as Y132F isolates), and most are clonally related (3–12). To date, clonal outbreaks of BSI caused by Y132F isolates have been reported in countries on four continents (3–12), with some isolates becoming endemic in the affected hospitals over several years (3, 9–12). The clonal transmission of Y132F isolates, fueled by environmental sources (such as nosocomial surfaces and the hands of health care workers) and the resistance of the isolates to environmental decontamination, may persist in hospitals (11, 12). Gradual replacement of fluconazole-susceptible (FS) BSI isolates by FNS isolates has resulted in much higher rates of FNS C. parapsilosis, ranging from 12% to 90%, in affected hospitals (4–14).

The clinical implications of phenotypes of C. parapsilosis are also largely unexplored, as are the mechanisms driving the increased clonal transmission of BSIs caused by FNS isolates. We recently detected two phenotypes (sinking and floating) among BSI isolates of C. parapsilosis during the performance of Clinical and Laboratory Standards Institute (CLSI) broth microdilution (BMD) antifungal susceptibility testing. Because two distinct phenotypes of C. parapsilosis isolates had not been described previously, here, we investigated the microbiological and molecular characteristics of C. parapsilosis BSI isolates with these two phenotypes and their role in the clonal transmission of C. parapsilosis BSI isolates harboring the *ERG11* mutation Y132F. We assessed all 197 C. parapsilosis BSI isolates collected over a 13-year period (2006 to 2018) at one tertiary-care hospital in South Korea, because FNS isolates of C. parapsilosis were persistently recovered in this hospital over a period of several years, as reported in our previous multicenter study (3).

## RESULTS

### Detection of the sinking phenotype.

**FIG 1:**
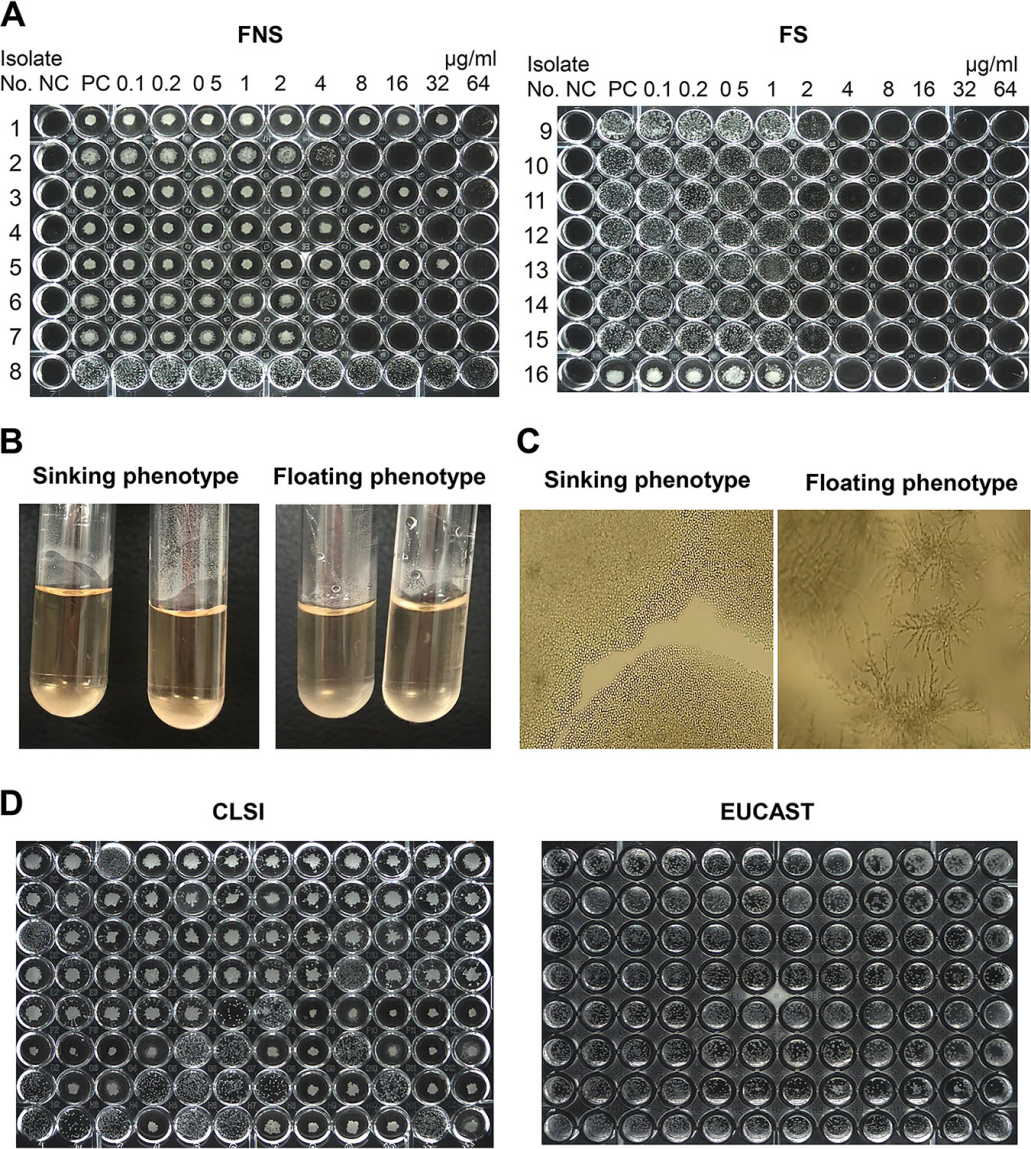
Detection of two phenotypes of C. parapsilosis. (A) Two representative phenotype patterns, i.e., sinking (isolates 1 to 7 and 16) and floating (isolates 8 to 15), of C. parapsilosis isolates, as determined using CLSI broth microdilution testing plates for testing fluconazole. Most of the FNS isolates exhibited a sinking phenotype (isolates 1 to 7) in CLSI U-shaped round-bottom wells after 48 h of incubation, whereas FS isolates usually exhibited floating phenotypes (isolates 8 to 15). NC, negative control; PC, positive control. (B) All yeast cells with the sinking phenotype sank to the bottom of the tubes, resulting in clear supernatants, but cells with floating phenotypes showed slightly turbid supernatants in tubes. (C) After 72 h of culture on cornmeal agar, all cells with the sinking phenotype were predominantly yeast cells without pseudohyphae or a few hyphae, whereas those with the floating phenotype produced abundant pseudohyphae. (D) Comparison of phenotype detection in the CLSI U-shaped round-bottom wells versus the EUCAST flat-bottom wells in 96-well plates.

The two phenotypes of C. parapsilosis was first detected by visual examination of the CLSI antifungal BMD plate wells after 48 h of incubation (Fig. 1A). Cells with the floating phenotype were dispersed in plate wells like other common *Candida* species isolates, whereas cells with the sinking phenotype had a smaller, button-like appearance. For each isolate, the phenotypic results obtained from CLSI susceptibility testing against six antifungal agents (fluconazole, voriconazole, amphotericin B, caspofungin, micafungin, and anidulafungin) were the same, as were those in the drug-free positive-control wells and the drug-containing (subinhibitory concentrations of antifungal agents) wells in the plate. All yeast cells with the sinking phenotype sank to the bottom of the tubes, resulting in clear supernatants, but cells with the floating phenotype showed slightly turbid supernatants in tubes (Fig. 1B). When plated on cornmeal-Tween 80 agar, all sinking phenotype isolates produced either no or only a few pseudohyphae, whereas all floating-phenotype cells produced pseudohyphae (Fig. 1C). In contrast to the CLSI microplates, the sinking and floating phenotypes could not be differentiated in the flat-bottom wells of the European Committee on Antimicrobial Susceptibility Testing (EUCAST) BMD microplates (Fig. 1D).

### Antifungal susceptibility, phenotyping, and ERG11 sequencing.

Among the 197 C. parapsilosis BSI isolates, 75 (38.1%) were FNS (36 fluconazole-resistant [FR] and 39 susceptible—dose dependent [F-SDD]) and 122 (61.9%) were FS (Table 1). Voriconazole resistance was detected in 15.5% (9/58), 9.1% (2/22), and 0% (0/118) of the FR, F-SDD, and FS isolates, respectively. None of the isolates were resistant to amphotericin B or three echinocandins. *ERG11* sequencing identified the Y132F substitution in 93.3% (70/75) of the FNS isolates. The sinking phenotype was observed in 88.9% (32/36), 84.6% (33/39), and 92.9% (65/70) of the FR, F-SDD, and Y132F isolates but in only 26.0% (33/127) and 27.0% (33/122) of the non-Y132F and FS isolates, respectively. Among 65 Y132F-sinking isolates, 32 (48.2%) were FR and 33 (50.8%) were F-SDD. Overall, 86.7% (65/75) of the FNS isolates and 49.7% (98/197) of all isolates exhibited the sinking phenotype. A comparison of the number of isolates between period A (2006 to 2013) and period B (2014 to 2018) showed a higher yearly isolation of FNS (2.4 versus 11.2 [period A versus period B]), Y132F (2.1 versus 10.6), and sinking (3.8 versus 13.6) isolates during period B. The numbers of sinking phenotype isolates harboring a Y132F *ERG11* mutation (referred to as Y132F-sinking isolates) during periods A and B were 2.1 and 9.6 per year, respectively.

**TABLE 1:** Result of fluconazole susceptibility testing, Erg11p sequencing, and phenotyping in 197 Candida parapsilosis bloodstream infection isolates from a university hospital during 2006 to 2018

Fluconazole susceptibility^*a*^	Erg11p sequencing phenotype	No. of C. parapsilosis bloodstream isolates isolated in yr													
		2006	2007	2008	2009	2010	2011	2012	2013	2014	2015	2016	2017	2018	Total (%)
FR	Y132F-sinking	1	1	1		2	2	1	2	5	2	4	3	8	32 (88.9)
	Y132F(+R398I)-floating													3	3 (8.3)
	Non-Y132F-floating												1		1 (2.8)
	Total	1	1	1	0	2	2	1	2	5	2	4	4	11	36 (100.0)
F-SDD	Y132F-sinking				1		1	3	2	4	4	6	9	3	33 (84.6)
	Y132F(+R398I)-floating													2	2 (5.1)
	Non-Y132F-floating			1					1				1	1	4 (10.3)
	Total	0	0	1	1	0	1	3	3	4	4	6	10	6	39 (100.0)
FS	Non-Y132F-sinking	3	1	3		2	1	3		5	6	2	5	2	33 (27.0)
	Non-Y132F-floating	6	5	13	1	4	6	6	8	12	6	7	12	3	89 (73.0)
	Total	9	6	16	1	6	7	9	8	17	12	9	17	5	122 (100.0)
Total	Y132F-sinking	1	1	1	1	2	3	4	4	9	6	10	12	11	65 (33.0)
	Y132F-floating	0	0	0	0	0	0	0	0	0	0	0	0	5	5 (2.5)
	Non-Y132F-sinking	3	1	3	0	2	1	3	0	5	6	2	5	2	33 (16.8)
	Non-Y132F-floating	6	5	14	1	4	6	6	9	12	6	7	14	4	94 (47.7)
	Total	10	7	18	2	8	10	13	13	26	18	19	31	22	197 (100.0)

a Fluconazole and interpretative categories of resistance were determined using the CLSI M27-4ED broth microdilution method and CLSI M60-ED, respectively (26, 27).

### Microsatellite typing.

**FIG 2:**
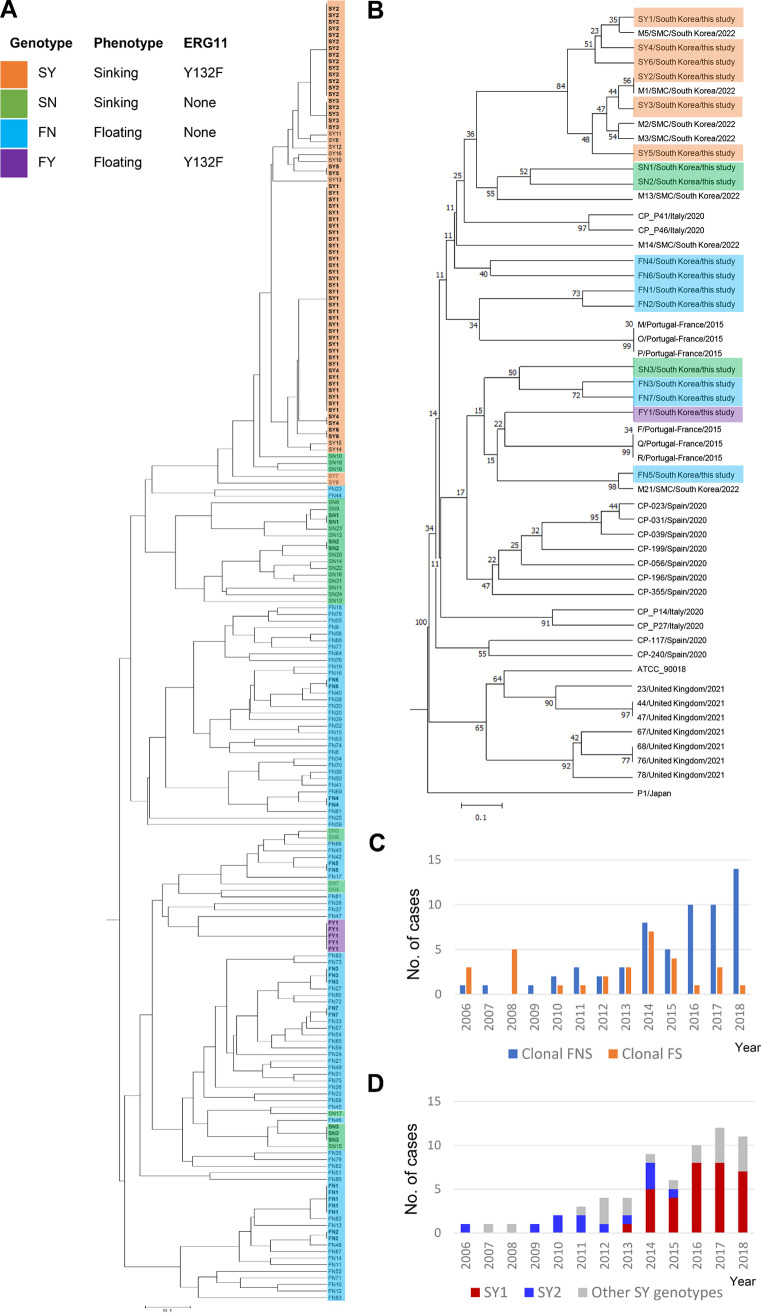
Microsatellite typing. (A) Dendrogram based on the microsatellite genotypes of all 197 bloodstream isolates obtained during 2006 to 2018. (B) Dendrogram comparing clonal genotypes of this study (SY1-6, SN1-3, FY1, and FN1-7) with those described in reports from several countries (genotype/country/publication year). Genotypes M, O, P, F, Q, and R are from Portugal/France (23); CP-P14, CP-P27, CP-P41, and CP-P46 are from Italy (24); 23, 44, 47, 67, 68, 76, and 78 are from the United Kingdom (25); P1 is from Japan (26); CP-023, 031, 056, 117, 196, and 240 are from Spain (27); CP-039 and 199 are from Italy (27); CP-355 is from Brazil (27); M1-3, M5, M13, M14, and M21 are from SMC in South Korea (14). (C) Yearly numbers of clonal FNS and clonal FS strains isolated. (D) Yearly numbers of Y132F-sinking isolates isolated, with their genotypes (33 SY1, 12 SY2, and 20 of other SY genotypes). Isolates from each of two dominant genotypes (SY1 and SY2) were persistently recovered for 6 and 10 years, respectively, and they accounted for 69.2% of all Y132F-sinking isolates.

Microsatellite typing of the 197 isolates yielded 124 different genotypes: 107 were unique to a single isolate, whereas 17 were shared by 90 isolates (45.7%) (referred to as clonal isolates) (Table 2). The sinking phenotype was more frequently observed in clonal isolates (74.4% [67/90]) than other isolates (29.0% [31/107]; *P < *0.0001). All isolates of the same clonal microsatellite genotype had the same (sinking or floating) phenotype. Among the clonal isolates, all 60 isolates with SY1-6 genotypes exhibited a sinking phenotype, and they had the Y132F mutation (with the exception of three SY2 and two SY3 isolates); all 7 isolates of SN1-3 had a sinking phenotype without the Y132F mutation, all 18 isolates of FN1-7 exhibited a floating phenotype without the Y132F mutation, and all 5 isolates of FY1 had a floating phenotype with the mutations Y132F and R398I. Clonality was observed more frequently for the sinking phenotype of C. parapsilosis (68.4% [67/98]) than for the floating phenotype (23.2% [23/99]; *P < *0.0001) and more frequently for the Y132F-sinking isolates (84.6% [55/65]) than for all other isolates (26.5% [35/132]; *P* < 0.0001). The microsatellite dendrogram of all 197 BSI isolates obtained at Severance Hospital during 2006 to 2018 shows that almost all Y132F-sinking isolates coclustered (Fig. 2A). When 17 clonal genotypes (genotypes SY1-6, SN1-3, FY1, and FN1-7) in this study were reanalyzed with the other 35 clonal genotypes reported from several countries, 10 (SY1-6, M1-3, and M5) genotypes of Y132F isolates from two Korean hospitals (Severance Hospital and Samsung Medical Center [SMC]) were found to be a distinct group without close relationships between clonal genotypes of the other countries (Fig. 2B). A comparison of the number of clonal isolates between period A (2006 to 2013) and period B (2014 to 2018) showed that clonal clusters of the FNS (*n *= 13) and FS (*n *= 15) isolates occurred at similar low rates during period A but clonal clusters of the FNS isolates (*n *= 47; mostly Y132F-sinking isolates) were more frequent than those of FS isolates (*n *= 16) during period B (Fig. 2C). Two dominant genotypes, SY1 and SY2, persistently recovered for 6 and 10 years, respectively, accounted for 69.2% (45/65) of all Y132F-sinking isolates (Fig. 2D).

**TABLE 2:** Results of microsatellite genotyping, phenotyping, Erg11p sequencing, and fluconazole susceptibility in 197 C. parapsilosis BSI isolates from a university hospital during 2006 to 2018

Microsatellite genotype^*a*^		Phenotype	Erg11p substitution(s)	No. (%) of C. parapsilosis isolates^*b*^			
Genotype	Allele profile			FR	F-SDD	FS	Total
SY1	240/240–306/336–264/282–127/133	Sinking	Y132F	10	23		33
SY2	240/246–306/306–264/282–127/127	Sinking	Y132F	8	4		12
SY2	240/246–306/306–264/282–127/127	Sinking	None			3	3
SY3	240/246–306/306–264/282–127/133	Sinking	Y132F	3			3
SY3	240/246–306/306–264/282–127/133	Sinking	None			2	2
SY4	240/240–306/336–264/288–127/133	Sinking	Y132F	1	2		3
SY5	246/246–306/306–282/282–127/127	Sinking	Y132F	2			2
SY6	240/240–306/336–264/282–107/127	Sinking	Y132F	2			2
SY7–SY12^*c*^		Sinking	Y132F	6			6
SY13–SY16^*c*^		Sinking	Y132F		4		4
SN1	243/243–306/306–258/258–151/151	Sinking	None			2	2
SN2	222/264–306/306–267/285–151/151	Sinking	None			2	2
SN3	225/246–282/369–270/294–129/155	Sinking	None			3	3
SN4–SN24^*c*^		Sinking	None			21	21
FY1	243/249–390/402–285/285–129/129	Floating	Y132F+R398I	3	2		5
FN1	243/243–354/354–273/273–103/103	Floating	None			5	5
FN2	243/243–351/351–273/273–103/103	Floating	None			2	2
FN3	243/243–369/369–210/210–129/129	Floating	None			3	3
FN4	246/249–420/423–273/294–115/127	Floating	None			2	2
FN5	210/243–309/309–306/306–129/129	Floating	None			2	2
FN6	246/246–375/423–288/288–125/125	Floating	None			2	2
FN7	246/246–369/369–210/210–129/129	Floating	None			2	2
FN8^*c*^		Floating	None	1			1
FN9–FN12^*c*^		Floating	None		4		4
FN13–FN83^*c*^		Floating	None			71	71
Subtotal	Clonal strains^*d*^	Sinking	Y132F	26	29	0	55
	Clonal strains^*d*^	Sinking	None	0	0	12	12
	Clonal strains^*d*^	Floating	Y132F	3	2	0	5
	Clonal strains^*d*^	Floating	None	0	0	18	18
	Nonclonal strains	Sinking	Y132F	6	4		10
	Nonclonal strains	Sinking	None			21	21
	Nonclonal strains	Floating	None	1	4	71	76
Total				36	39	122	197

a For microsatellite typing, each strain was characterized by a genotype resulting from the combination of the sizes of the four markers (CP1, CP4, CP6, and B) (3, 14, 23–27).

b Antifungal susceptibility and interpretative categories of resistance were determined using the CLSI M27-4ED broth microdilution method and CLSI M60-ED, respectively (26, 27). FR, fluconazole resistant (MIC, ≥8 μg/mL); F-SDD, fluconazole susceptible—dose dependent (MIC, 4 μg/mL); FS, fluconazole susceptible (MIC, ≤2 μg/mL).

c Genotypes (total of 107 isolates) derived from a single isolate.

d Clonal strains were defined by the isolation of an identical microsatellite genotype from more than one patient.

### Clinical characteristics.

The overall crude 30-day rate of mortality due to C. parapsilosis BSI was 27.4%. The crude 30-day rates of mortality from BSI caused by Y132F-sinking, non-Y132F-sinking, Y132F-floating, and non-Y132F-floating isolates were 32.3%, 21.2%, 20.0%, and 26.6%, respectively; the differences were not significant. Table 3 summarizes the results of a baseline characteristics of patients with C. parapsilosis BSIs and the risk factors for BSIs with Y132F-sinking isolates assessed by uni- and multivariate logistic regression analyses. Several characteristics were more frequently found in the patients with candidemia due to Y132F-sinking isolates, including vasopressor use, multifocal colonization, intensive care unit (ICU) admission, concomitant bacteremia, urine catheter use, central venous catheter (CVC) use, prior antifungal exposure (especially for azole), and breakthrough fungemia (especially for azole). In a multivariate analysis, azole breakthrough fungemia (odds ratio [OR], 6.540), ICU admission (OR, 5.044), and urinary catheter placement (OR, 6.918) were independent risk factors for candidemia caused by Y132F-sinking isolates.

**TABLE 3:** Baseline clinical characteristics of patients with C. parapsilosis BSIs and independent risk factors for BSIs with Y132F-sinking isolates, determined by uni- and multivariate logistic regression analyses

Characteristic^*a*^	Value (%) for patients with:			*P*	Odd ratio (95% confidence interval)	
	All isolates (*n* = 197)	Y132F-sinking isolates (*n* = 65)	All others (*n* = 132)			
					Univariate analysis^*b*^	Multivariate analysis^*c*^
Demographics and comorbidities						
Female	33.5	23 (35.4)	43 (32.6)	0.749		
Diabetes mellitus	27.9	18 (27.7)	37 (28)	1		
Liver diseases	10.2	6 (9.2)	14 (10.6)	1		
Chronic kidney diseases	19.3	18 (27.7)	20 (15.2)	0.054	2.145 (1.042–4.416)	
Congestive heart failure	6.6	3 (4.6)	10 (7.6)	0.551		
Myocardial infarction	11.2	7 (10.8)	15 (11.4)	1		
COPD	9.6	8 (12.3)	11 (8.3)	0.443		
Peripheral vascular disease	6.6	7 (10.8)	6 (4.5)	0.127		
Cerebrovascular disease	19.8	17 (26.2)	22 (16.7)	0.13		
Dementia	7.6	5 (7.7)	10 (7.6)	1		
Hemiplegia	15.2	14 (21.5)	16 (12.1)	0.094	1.99 (0.904–4.382)	
Peptic ulcer disease	18.8	16 (24.6)	21 (15.9)	0.174	1.726 (0.83–3.589)	
Connective tissue disease	4.1	5 (7.7)	3 (2.3)	0.118	3.583 (0.829–15.488)	
Solid tumor	43.7	21 (32.3)	65 (49.2)	0.032	0.492 (0.264–0.916)	
Leukemia	2.5	1 (1.5)	4 (3)	1		
Lymphoma	1.0	0 (0)	2 (1.5)	1		
Vasopressor use	50.8	48 (73.8)	52 (39.4)	<0.001	4.344 (2.258–8.355)	
Acute kidney injury	34.5	27 (41.5)	41 (31.1)	0.155		
High ACCI	58.9	36 (55.4)	80 (60.6)	0.539		
High *Candida* score	3.0	2 (3.1)	4 (3)	1		
Clinical status at positive culture		(0)	(0)			
Total parenteral nutrition	91.4	59 (90.8)	121 (91.7)	0.794		
Prior surgery	23.9	17 (26.2)	30 (22.7)	0.598		
Severe sepsis	10.2	5 (7.7)	15 (11.4)	0.616		
Multifocal colonization	6.6	8 (12.3)	5 (3.8)	0.033	3.565 (1.117–11.374)	
Neutropenia	3.6	2 (3.1)	5 (3.8)	1		
Immunosuppressive therapy	9.6	10 (15.4)	9 (6.8)	0.072	2.485 (0.956–6.457)	
Intensive care unit	57.4	55 (84.6)	58 (43.9)	<0.001	7.017 (3.294–14.951)	5.044 (2.252–11.297)
Concomitant bacteremia	13.2	14 (21.5)	12 (9.1)	0.024	2.745 (1.188–6.344)	
Urine catheter use	70.6	60 (92.3)	79 (59.8)	<0.001	8.051 (3.032–21.374)	6.918 (2.391–20.014)
CVC	89.3	64 (98.5)	112 (84.8)	0.003	11.429 (1.498–87.167)	
Prior antifungal exposure	19.3	23 (35.4)	15 (11.4)	<0.001	4.271 (2.038–8.952)	
Prior azole exposure	14.7	18 (27.7)	11 (8.3)	0.001	4.213 (1.851–9.587)	
Prior echinocandin exposure	4.6	5 (7.7)	4 (3)	0.159		
Prior AMB exposure	4.6	5 (7.7)	4 (3)	0.159		
All breakthrough fungemia	11.7	15 (23.1)	8 (6.1)	0.001	4.65 (1.855–11.654)	
Azole breakthrough fungemia	9.1	13 (20)	5 (3.8)	<0.001	6.35 (2.155–18.712)	6.54 (1.803–23.725)
Echinocandin breakthrough fungemia	2.0	2 (3.1)	2 (1.5)	0.6		
AMB breakthrough fungemia	0.5	0 (0)	1 (0.8)	1		

a COPD, chronic obstructive pulmonary disease; ACCI, age-adjusted Charlson comorbidity index; CVC, central venous catheter; AMB, amphotericin B.

b The results of univariate analysis are listed only for variables that were statistically significant (*P* < 0.05).

c The potential predictive factors in the univariable comparisons (*P *< 0.1) were included in an initial multivariable regression model. The results of multivariate analysis are listed for results with statistical significance (*P* < 0.05).

### Microbiological characteristics and virulence traits.

**FIG 3:**
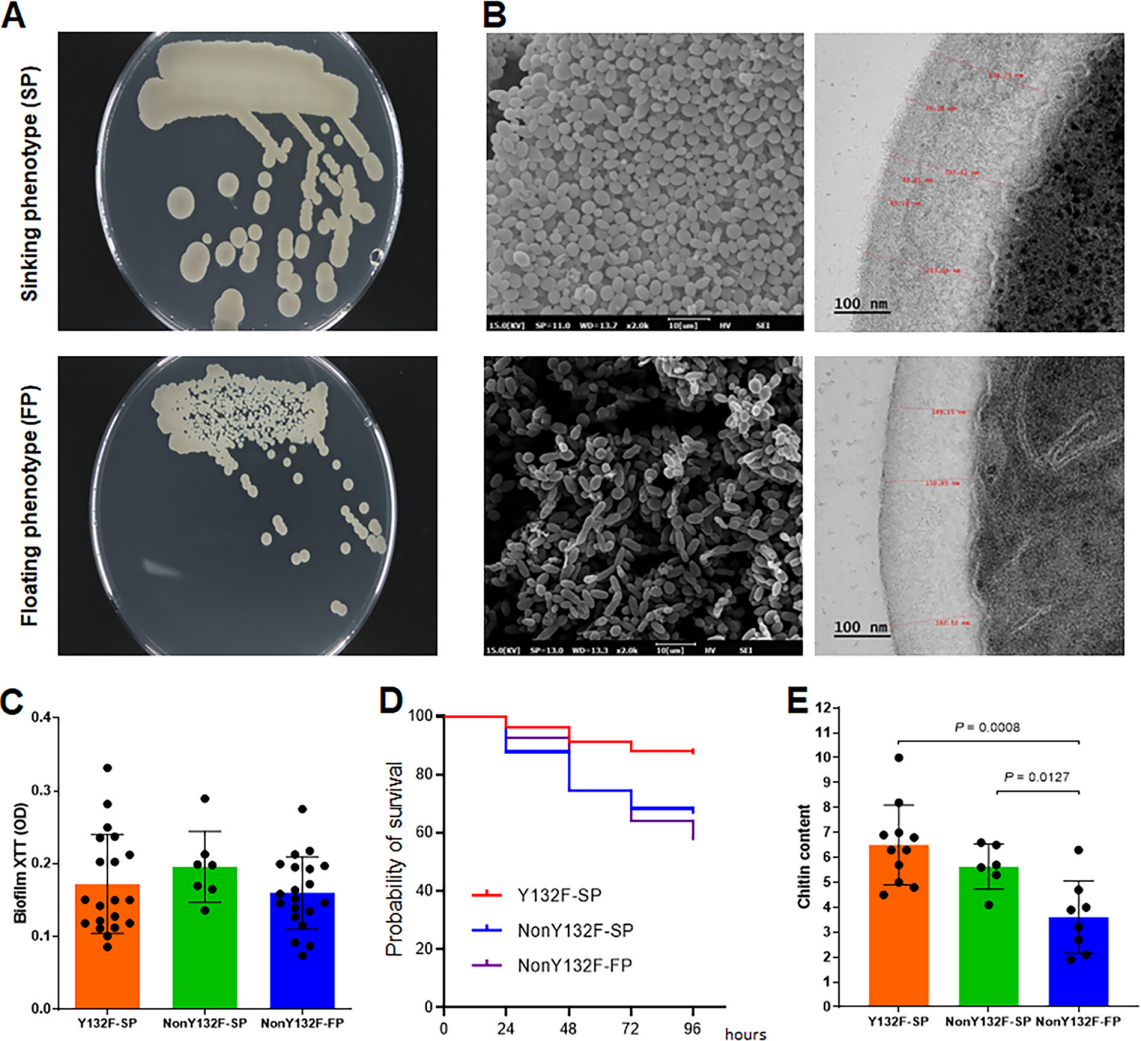
Microbiological characteristics and virulence traits of sinking phenotype. (A) Sinking isolates exhibited larger colonies with a smooth margin on PDA (48-h growth). (B) Electron microscopic images of sinking phenotype isolates reveal many spherical cells without pseudohyphae and a thicker cell wall compared with floating phenotype isolates. (C) Mean biofilm-forming abilities, assessed using the XTT reduction assay, were not significantly different among the three isolate groups. OD, optical density. (D) The mean survival rate of larvae infected with Y132F-sinking isolates was significantly higher than that of larvae infected with non-Y132F-sinking or non-Y132F-floating isolates. (E) Mean chitin contents of Y132F-sinking and non-Y132F-sinking isolates were significantly higher than that of floating isolates, as determined in a flow cytometric assay based on calcofluor white staining.

When plated on potato dextrose agar (PDA), cells with a sinking phenotype formed mostly large smooth colonies with round margins, whereas the colony size of those with a floating phenotype varied, with most colonies having irregular margins (Fig. 3A). Electron microscopy of sinking-phenotype isolates revealed spherical cells that were stacked but did not stick to each other and a cell wall that was thicker (median, 209.8 nm; range, 148.9 to 314.3 nm) than that of the floating cells (median, 163.9 nm; range, 131.8 to 186.2 nm) (*P < *0.0001) (Fig. 3B). There were no significant differences in the mean biofilm-forming ability among Y132F-sinking, non-Y132F-sinking, and non-Y132F-floating isolates (Fig. 3C). The mean survival rate of Galleria mellonella larvae infected with Y132F-sinking isolates was significantly higher than that of larvae infected with non-Y132F-sinking isolates (log-rank test, hazard ratio [HR] of Y132F-sinking versus non-Y132F-sinking, 0.3477 [95% confidence interval [CI], 0.2268 to 0.5530], *P < *0.0001) or non-Y132F-floating isolates (log-rank test, HR of Y132F-sinking versus non-Y132F-floating, 0.2858 [95% CI, 0.1989 to 0.4106], *P < *0.0001) (Fig. 3D). When the relative chitin content was measured using a flow-cytometric assay based on calcofluor white staining, the mean (standard deviation [SD]) fluorescence intensity of Y132F-sinking (6.500 ± 1.597) and non-Y132F-sinking (5.633 ± 0.9114) isolates was significantly higher than non-Y132F-floating isolates (3.600 ± 1.457) (Mann-Whitney test, all sinking versus non-Y132F-floating, *P = *0.0003; Y132F-sinking versus non-Y132F-floating, *P = *0.0008; nonY132F-sinking versus non-Y132F-floating, *P = *0.0127) (Fig. 3E).

## DISCUSSION

Our study is the first to show that long-term clonal transmission of C. parapsilosis BSI isolates harboring a Y132F *ERG11* mutation in Korean hospitals involved isolates with sinking phenotypes, which are quite different from those with floating phenotypes. Unlike isolates with the floating phenotype, those with the sinking phenotype were easily detected by their smaller button-like appearance in CLSI antifungal BMD plate wells, because all yeast cells sank to the round bottoms of the U-shaped wells. All isolates of the same microsatellite genotype had the same (sinking or floating) phenotype, suggesting that phenotype may align with the genotype of those isolates. Almost all Y132F-sinking isolates were clonally related, and two dominant clonal genotypes persistently recovered over 6 or 10 years accounted for 69.2% of all Y132F-sinking isolates. Given that the sinking phenotype was observed predominantly in Y132F (91.5%), FNS (86.7%), and clonal (74.4%) BSI isolates of C. parapsilosis and that azole breakthrough fungemia, ICU admission, and urinary catheter use were independent risk factors for fungemia caused by Y132F-sinking isolates, we postulated that strains with a sinking phenotype may be prone to having an *ERG11* Y132F substitution after azole exposure, may be more enriched, and may persist for a long time in the ICU environment, where they may cause BSIs in vulnerable patients with indwelling catheters or azole exposure.

Kuhn et al. found that clonal isolates of C. parapsilosis had a higher ability to form biofilms than did unrelated strains, suggesting that biofilm production plays a role in C. parapsilosis outbreaks and provides an index of persistence in the hospital setting (15). In contrast, Thomaz et al. reported a low biofilm-forming ability of all clonal Y132F isolates of C. parapsilosis (7). We did not find any significant differences in biofilm formation between clonally related Y132F-sinking and other nonclonal isolates, indicating that biofilm formation is not a critical causal factor of clonal transmission or outbreaks caused by FNS isolates of C. parapsilosis. However, we did note that clonality was more frequent among sinking (68.4%) than among floating (23.2%) phenotypes of C. parapsilosis. In addition, cells with the sinking phenotype were considerably different from cells with the floating phenotype. The microbiological characteristics of the sinking isolates, including colony morphology and cornmeal agar morphology, resemble those previously reported for smooth-colony isolates (16), but we could not differentiate sinking from floating phenotypes based on colony morphology alone. Our results therefore suggest that the unique characteristics of the sinking phenotype likely enable this yeast to survive in the presence of multiple stressors, such as environmental pressure and antifungal exposure, which plays an important role in C. parapsilosis regarding clonal spread, rather than biofilm formation.

Recent studies have shown that BSIs caused by FR Y132F isolates of C. parapsilosis are associated with a higher 30-day mortality (50.0% or 63.8%) (9, 11). In the present study, the Y132F-sinking isolates exhibited both FR (48.2%) and F-SDD (50.8%), but there was no statistical difference in the 30-day mortality of patients with BSIs cause by Y132F-sinking (32.3%) versus by other (24.8%) isolates, which may be partly due to the lower virulence of Y132F-sinking isolates. In the present study, in comparison with floating phenotype isolates, the Y132F-sinking phenotypes of C. parapsilosis formed fewer pseudohyphae, considered a major virulence factor (17), and were less virulent than other isolates in the G. mellonella model, in agreement with a recent report (18). In this study, Y132F-sinking BSI isolates were significantly associated with ICU admission and vasopressor use, although the latter was not an independent risk factor for BSIs caused by Y132F-sinking isolates. Considering their lower virulence, Y132F-sinking isolates may not frequently cause severe fungemia, which results in hemodynamic instability and vasopressor use in ICU patients. Alternatively, the long-term persistence of Y132F-sinking strains in the hospital environment may cause BSIs in vulnerable patients on vasopressor therapy in ICUs, where vasopressors are typically administered via a CVC (19).

The increase in the prevalence of FR C. parapsilosis isolates has been a major threat in developing countries, in which the vast majority of candidemia cases are treated with fluconazole (1). However, a recent Korean single-center study showed a significant increase in FR C. parapsilosis candidemia in the period with an increased echinocandin use for candidemia treatment (35.3% versus 0.0%, respectively) after the approval of echinocandin use by the National Health Insurance Service in 2014 in Korea (20). Similarly, we also found increases in FNS (4.7-fold), Y132F (5.1-fold), sinking (3.6-fold), and Y132F-sinking (4.6-fold) isolates of C. parapsilosis after 2014, which were paralleled by an increase in the number of candidemia patients who received echinocandin therapy from 2014 at Severance Hospital, as reported in a previous study (21). All of these data suggest that Y132F-sinking isolates of C. parapsilosis continue to be a nosocomial threat in the era of echinocandin therapy.

Unlike other common *Candida* species, C. parapsilosis has a naturally occurring amino acid substitution, P660A, in the *FKS1* hot spot 1 region, which might reduce susceptibility to echinocandins *in vitro* (17). In this study, sinking isolates had significantly higher chitin contents than floating isolates. In addition, the walls of sinking isolates were thicker than those of floating isolates, although electron micrographs of only a few isolates were obtained. The thicker wall and elevated wall chitin content have been found in Candida albicans isolates with *FKS* hot spot mutations, which show reduced fitness and attenuated pathogenicity (22, 23). *Candida* populations that survive echinocandin exposure develop tolerance to echinocandin agents and have a high chitin content in the cell wall (24, 25). Further studies are needed to elucidate the factors contributing to the recent rise in incidence of BSIs due to Y132F-sinking isolates of C. parapsilosis in Korea and to evaluate whether the selection of Y132F-sinking subpopulations of C. parapsilosis coincides with echinocandin introduction.

The present study demonstrates that the same clonal Y132F-sinking isolates were persistently recovered in two hospitals (Severance Hospital and SMC) in South Korea over several years, which was consistent with our previous multicenter study (3). A recent study performed at the SMC demonstrated the continued evolution of azole resistance mechanisms (*MRR* gene mutation) and thus increasing fluconazole MICs over time in clonal Y132F isolates (14). Further increases in the fluconazole MICs of these clonal Y132F-sinking isolates may pose significant challenges to antifungal management, with an increasing rate of azole breakthrough fungemia. Therefore, we suggest that the detection of the sinking phenotype of FNS C. parapsilosis BSI isolates serves as an indicator of ongoing undetected long-term clonal transfer of Y132F-sinking isolates within a hospital.

## MATERIALS AND METHODS

### Fungal isolates, antifungal susceptibility testing, phenotyping, and molecular analysis.

From January 2006 to December 2018, 197 nonduplicate C. parapsilosis BSI isolates were collected at Severance Hospital (2,400 beds; Seoul, South Korea). The *Candida* isolates were identified to the species level using matrix-assisted laser desorption ionization–time of flight mass spectrometry and/or molecular methods (3, 4). CLSI M27-A3 BMD tests for susceptibility to fluconazole, voriconazole, amphotericin B, caspofungin, micafungin, and anidulafungin were performed, and the interpretative guidelines in the CLSI M60-ED1 document were used to classify the isolates based on clinical breakpoints (26, 27). Two phenotypes (sinking and floating) were determined by visually examining the CLSI BMD plate wells after incubating the isolates, as well as by assessing the morphology of the yeasts on cornmeal-Tween 80 agar. *ERG11* was sequenced for all isolates as described previously (3). Microsatellite typing using four markers (CP1, CP4, CP6, and B) was performed for genotype determination also as described previously (3, 14, 28–32). Clonal strains were defined by the isolation of an identical microsatellite genotype from more than one patient. We further evaluated the genetic relationships of genotypes of clonal strains from our cohort with 35 genotypes of clonal strains available in the previous publications (14, 28–32). Microsatellite dendrograms were constructed using the unweighted pair-group method with arithmetic mean (UPGMA), as described elsewhere (33).

### Clinical data collection.

The collected data included patient demographics, comorbidities, severity of infection, clinical status at the time of positive culture, and therapeutic measures (34–36). Breakthrough fungemia was the development of candidemia during antifungal therapy (34–37). This study was approved by the Institutional Review Board of Yonsei University College of Medicine (YUHS 4–2019–0970).

### Microbiological and virulence trait study.

Colony morphology on PDA was examined for all isolates. The cell surface topography and cell wall morphology of four selected isolates (three sinking and one floating phenotypes) were visualized using electron microscopy, as described previously (38). Biofilm formation was assessed using an XTT [2,3-bis-(2-methoxy-4-nitro-5-sulfophenyl)-2H-tetrazolium-5-carboxanilide salt] reduction assay (39), and *in vivo* virulence was studied using the insect G. mellonella (40) for 25 selected isolates (11 Y132F-sinking, 6 non-Y132F-sinking, and 8 non-Y132F-floating isolates). In addition, the chitin contents of these 25 isolates were measured using a flow-cytometric assay based on calcofluor white staining, as reported previously (41).

### Statistical analyses.

SPSS 27.0 (IBM Corp., Armonk, NY, USA) was used for the statistical analysis and Prism 9.3.1 (GraphPad Software Inc., San Diego, CA, USA) for the graphical work. Univariate analyses were based on the chi-squared or Fisher’s exact test, as appropriate, for discrete variables. The predictive factors for C. parapsilosis fungemia caused by Y132F-sinking isolates were analyzed using univariate and multivariate logistic regression analyses. Potential predictive factors in the univariable comparisons (*P < *0.1) were included in an initial multivariable regression model. *P* values of <0.05 were considered statistically significant.
